# A Man With Recurrent Pneumonitis: A Rare Case of Interstitial Lung Disease Associated With Anti-Mi-2 Beta-Specific Dermatomyositis

**DOI:** 10.7759/cureus.20334

**Published:** 2021-12-10

**Authors:** Anam Ahmad, Yeswanth Attoti, Keith A Bernstein

**Affiliations:** 1 Internal Medicine, St. Luke's Hospital, Chesterfield, USA

**Keywords:** anti-mi-2 antibody, steroid-sparing agent, dermatomyositis, interstitial lung disease, pneumonitis, myositis, steroids

## Abstract

Dermatomyositis (DM) and polymyositis (PM) are idiopathic inflammatory myopathies. Interstitial lung disease (ILD) develops in most patients with DM and PM directly related to morbidity and mortality. Diagnosis requires a myositis panel and high-resolution computed tomography (HRCT). Prognosis depends on specific myositis-specific antibodies and the pattern of the interstitial lung changes. Anti-Mi-2 antibody-specific dermatomyositis has a lower prevalence of interstitial lung disease and has a favorable prognosis, responding well to steroids. Our patient is a 72-year old male who presented with recurrent episodes of pneumonitis, and ILD was found to have anti-Mi-2 beta-specific dermatomyositis and SLE overlap disease. He was responding well to high-dose steroids but was rebounding to similar symptoms whenever steroid weaning was attempted. He was started on azathioprine, but unfortunately, his disease rapidly progressed, and he died within a few months. This manuscript enhances the temporal relationship between dermatomyositis and ILD.

## Introduction

Dermatomyositis (DM) and polymyositis (PM) are idiopathic inflammatory myopathies with an incidence of one in 100,00 persons. The incidence of interstitial lung disease (ILD) is 30 per 10,000 in the USA overall. ILD develops in 20%-80% of patients with DM and PM, causing significant morbidity and mortality. Symptoms range from a dry cough to progressive respiratory failure. Anti-Mi-2 antibody-specific dermatomyositis is a subgroup of DM with a lower prevalence of interstitial lung disease and malignancy [1]. We report an unusual case of organizing pneumonia associated with anti-Mi-2 beta-specific dermatomyositis and SLE overlap.

## Case presentation

**Figure 1:**
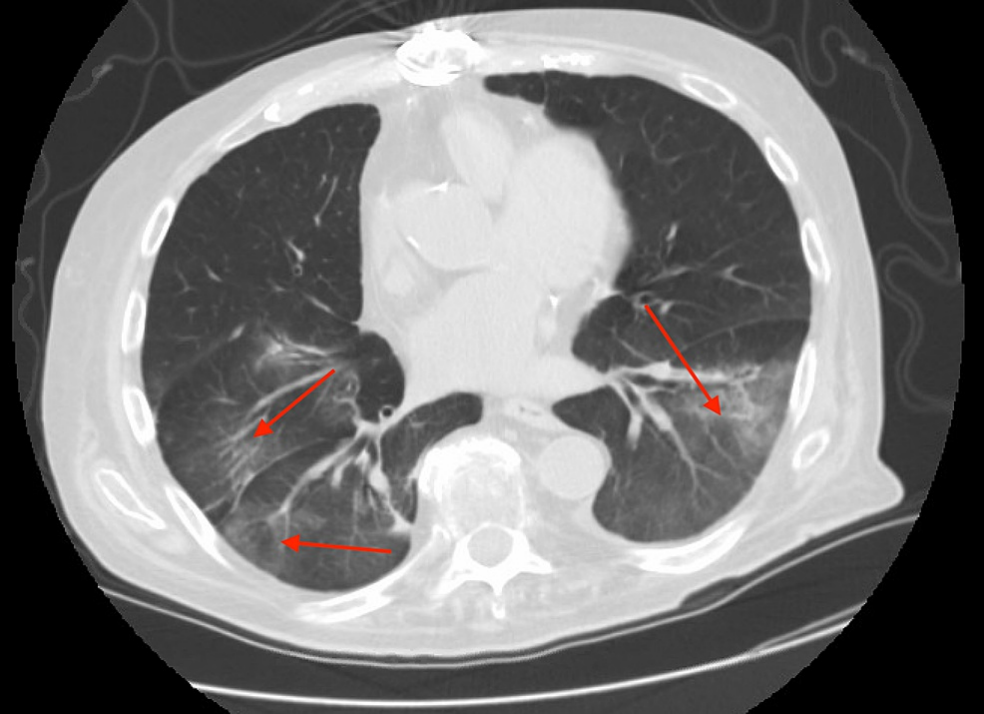
Chest CT showing patchy bilateral ground-glass infiltrates (arrows)

Our patient is a 72-year-old male with a history of congestive heart failure, coronary artery disease, hypertension, hyperlipidemia, cardiovascular accident, and atrial fibrillation treated with amiodarone (200 mg a day). He was being admitted to the medical floor for dry cough and shortness of breath. His initial vitals were unremarkable with a temperature of 35.8°C, pulse of 75 beats/minute, respiratory rate of 16 breaths/minute, SaO_2_ of 100% on room air, and blood pressure of 121/80 mmHg. Physical examination was significant of coarse crackles in the lungs with decreased air entry. Laboratory tests including complete blood count, complete metabolic profile, and B-type natriuretic peptide were unremarkable. The chest X-ray findings were consistent with bibasilar infiltrates. Subsequent chest computed tomography (CT) showed patchy ground-glass interstitial infiltrates (Figure 1). The patient had a negative multiplex respiratory PCR panel, including COVID-19 PCR and negative sputum culture. However, he was treated with empirical antibiotics, and amiodarone was also stopped for presumed ILD from the drug, as per high clinical suspicion. Bronchoscopy and biopsy were not considered due to his multiple comorbid conditions. The patient was given a tapered dose of steroids. He initially responded well to the steroids but eventually rebounded to similar symptoms whenever steroid weaning was attempted. 

The patient's dry cough and shortness of breath persisted, leading to recurrent hospital admissions. Also, in a couple of months, the patient started noticing progressive weakness in his proximal muscles, more pronounced in the lower extremities without any significant sensory loss leading to recurrent falls.

**Figure 2:**
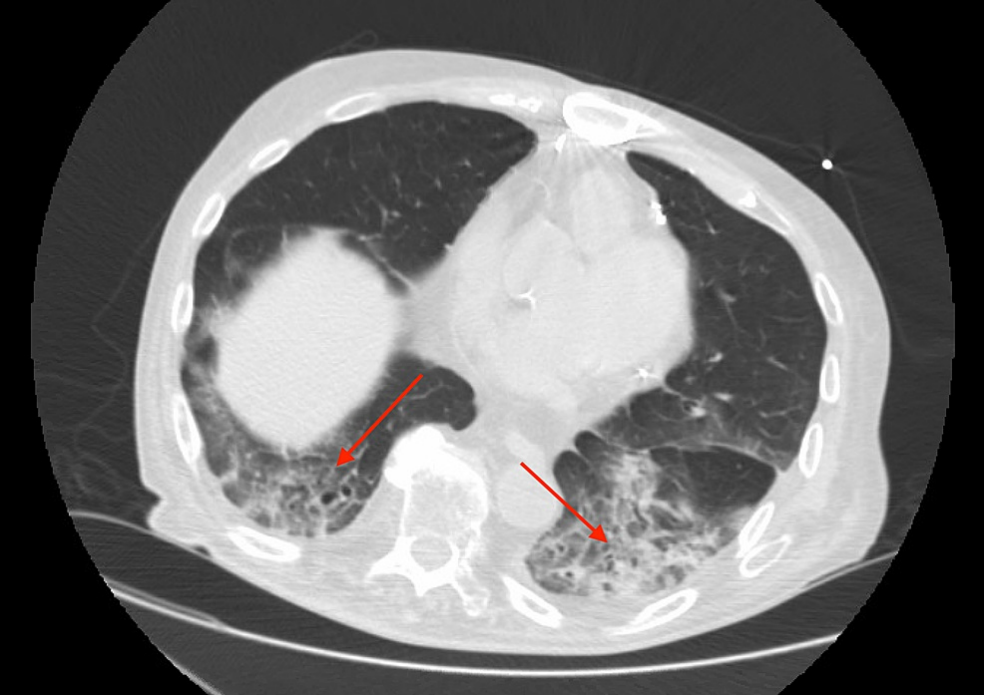
Chest CT showing worsening of infiltrates, raising suspicion of organizing pneumonia (arrows)

This constellation of persistent symptoms, including muscle weakness and ILD despite adequate treatment, led to the suspicion of a possible rheumatological pathology prompting workup. He was found to have elevated C-reactive protein (CRP), positive ANA with the titer of 1:80 with centromere pattern, low complement (C3 and C4) levels, and positive anti-dsDNA of 11 IU, suggestive of SLE. The myositis panel showed positive anti-Mi-2 beta antibodies, which led to the diagnosis of dermatomyositis. Repeat CT scan findings showed increased patchy bibasilar ground-glass infiltrates suggestive of pneumonitis and organizing pneumonia (Figure 2).

**Figure 3:**
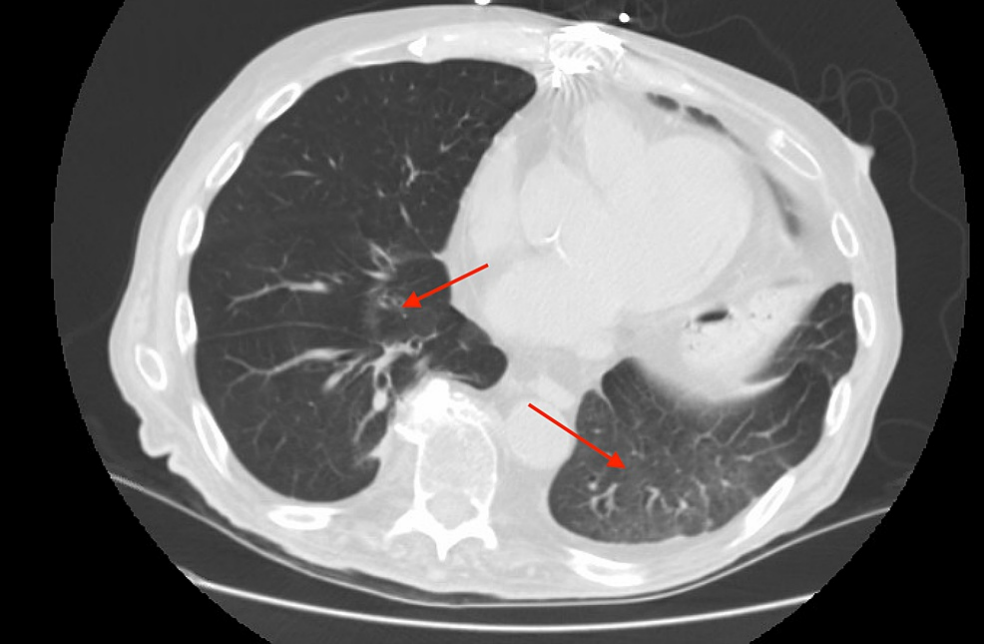
Repeat chest CT after three months of treatment showing improvement of infiltrates (arrows)

He was started on azathioprine and steroids, showing gradual improvement in his symptoms and CT scan findings (Figure 3).

## Discussion

Dermatomyositis is a systemic autoimmune myopathy that generally affects the skin and skeletal muscles [2]. Pathogenesis involves microangiopathy affecting mainly the skin and muscle fibers [3]. Myositis-specific antibodies such as anti-synthetase antibodies, anti-Mi-2, melanoma differentiation-associated gene 5 (MDA5) antibodies, and anti-aminoacyl tRNA synthetase (ARS) antibodies help identify the clinical subset of dermatomyositis and its prognosis [4].

Anti-Mi-2 antibodies are against helicase involved in transcriptional activation, strongly associated with dermatomyositis, and present in around 20% of patients with DM. They have a sensitivity of 4%-18% and a specificity of 98%-100%. Anti-Mi-2 antibody dermatomyositis is typically associated with skin lesions (V sign, shawl sign, and Gottron papules) absent in our patients. Interstitial lung disease is uncommon in patients with anti-Mi-2-specific dermatomyositis [5].

The suspicion for ILD associated with dermatomyositis begins when a patient with known DM and PM develops radiographic features related to ILD or if the patient who already has a clinical picture of interstitial lung disease develops features of myopathy, as in our patient [6]. Other differentials that can include infections, drug-related pneumonitis, primary diseases such as sarcoidosis, vasculitis, and other connective tissue disorders that can cause ILD have to be ruled out. 

Thus, the initial evaluation should include a complete blood count, comprehensive metabolic profile, and N-terminal pro-BNP level. Elevated ferritin, C-reactive proteins, and erythrocyte sedimentation rate can identify an underlying inflammatory process. If the diagnosis of DM or PM is suspected, we obtain creatinine kinase and aldolase levels, antinuclear antibody panel, anti-Jo-1 antibody, and myositis panel. Generally, patients will have a high serum level of muscle enzymes at the onset of the disease, indicating severe muscle disease. High-resolution computed tomography (HRCT) has high sensitivity and specificity to determine the extent and pattern of interstitial changes [7]. Pulmonary function tests are helpful to assess the progression of the disease [8]. Lung biopsy is helpful, but not required for the diagnosis.

The initial management of ILD in dermatomyositis is glucocorticoids, followed by glucocorticoid-sparing agents. Young patients with predominant polymyositis feature with CT scan findings consistent with nonspecific interstitial pneumonia respond well to steroids. Steroid-sparing agents are typically added in settings when patients have progressive disease on presentation or respond well to steroids but cannot taper or become intolerant to side effects. The commonly used agents are azathioprine, mycophenolate, and methotrexate for mild to moderate disease. For refractory cases, rituximab, cyclophosphamide, and IVIG are more effective [9]. Patients with anti-Mi-2 antibody DM generally respond very well to steroids.

The prognosis of ILD related to dermatomyositis depends on myositis-specific antibodies and the pattern of interstitial changes. Anti-Mi-2 antibody dermatomyositis has favorable outcomes with a low occurrence of pulmonary involvement [10]. Dermatomyositis, in general, is strongly associated with a wide range of cancers, but anti-Mi-2 antibody DM has a rare association with malignancy [10].

Our patient presented with recurrent pneumonitis, muscle weakness, generalized fatigue, and deconditioning. His recovery was relatively slow, and it was a challenge to tapper him off the steroids. In our opinion, the unique feature of our case was the presence of both anti-Mi-2 beta and anti-dsDNA antibodies. This unique antibody combination suggested an overlap of dermatomyositis and systemic SLE, leading to this challenge and slow recovery. 

## Conclusions

The temporal relationship between the onset of interstitial lung disease and myositis is variable. Anti-Mi-2 antibody myositis usually presents with skin rash and muscle weakness. Its association with ILD is very uncommon but has favorable outcomes. Our patient is a rare case of DM with positive anti-Mi-2 antibodies whose initial manifestation was ILD; overlap with SLE made the case unique. Physicians should be aware of unusual presentations of dermatomyositis as prompt diagnosis helps them in proper management, leading to delayed morbidity and perhaps better overall prognosis.
